# Development of Real-Time PCR Based on *A137R* Gene for the Detection of African Swine Fever Virus

**DOI:** 10.3389/fvets.2021.753967

**Published:** 2021-11-29

**Authors:** Dan Yin, Renhao Geng, Hui Lv, Chunhui Bao, Hongxia Shao, Jianqiang Ye, Kun Qian, Aijian Qin

**Affiliations:** ^1^The International Joint Laboratory for Cooperation in Agriculture and Agricultural Product Safety, Ministry of Education, Yangzhou University, Yangzhou, China; ^2^Jiangsu Co-innovation Center for Prevention and Control of Important Animal Infectious Diseases and Zoonoses, Yangzhou, China; ^3^Jiangsu Key Laboratory of Zoonosis, Yangzhou, China

**Keywords:** African swine fever virus, *A137R* gene, SYBR Green, real-time PCR, detection

## Abstract

African swine fever virus (ASFV) can infect domestic pigs and wild boars and causes huge economic losses in global swine industry. Therefore, early diagnosis of ASFV is important for the control and eradication of African swine fever (ASF). In this study, a SYBR Green-based real-time polymerase chain reaction (PCR) assay targeting the viral encoded *A137R* gene was established for the detection of ASFV infection. For the evaluation of the established real-time PCR, 34 clinical samples were assessed by both the *A137R* gene-based real-time PCR and OIE-recommended TaqMan PCR. The results showed that 85.29% (29/34) were detected by *A137R* gene-based real-time PCR, but only 79.41% (27/34) positive using OIE-recommended TaqMan PCR. Moreover, no cross-reaction with other common swine pathogens was found in the *A137R* gene-based real-time PCR. These results demonstrated that the established real-time PCR assay in this study showed better performance than the OIE-recommended method in detecting ASFV from clinical samples, which could be applied for control and eradication programs of ASF.

## Introduction

African swine fever (ASF) is a contagious viral disease characterized by fever, cutaneous congestion, multiple hemorrhages in the internal organs, ataxia, severe depression, and high mortality in acute cases. The virus affects pigs of all species, breeds, and ages (1). In August 2018, ASF was reported for the first time in China, which heralded a new transmission era. Subsequently, ASF spreads to the entire Southeast Asia in the next year (2–4). Currently, the outbreaks of ASF are still ongoing in Africa, the trans-Caucasus region, Eastern Europe, Russian Federation and Asia, which caused a huge challenge to the swine industry in these regions (4).

The improvement of African swine fever virus (ASFV) diagnostic methods is one of the essential steps to control and prevent the ASF. Up to date, there is no commercial vaccine for ASF. Moreover, considering the similarities in clinical symptoms between ASF and other swine diseases, such as classical swine fever (CSF), and the potential appearance of non-specific clinical symptoms, the rapid and reliable diagnosis is essential for timely implementation of control measures to block the spread of this devastating disease (5, 6). In World Organization for Animal Health (OIE) handbook, the common laboratory diagnostic methods of ASFV include virus isolation (VI), the haemadsorption (HAD) test, polymerase chain reaction (PCR), loop-mediated isothermal amplification (LAMP), recombinase polymerase amplification (RPA), enzyme-linked immunosorbent assay (ELISA), immunoblotting test and indirect immunofluorescent assay (IFA) (7–9). Virus isolation and HAD using porcine macrophages cells are prestigious methods for ASFV diagnosis. But both methods require excellent laboratory skills and more than 6 days to get the result (10, 11). The conventional PCR recommended by OIE showed lower sensitivity when detecting field and experimental samples infected with vp72 genotype II strains, which may be due to a nucleotide mismatch between primer and viral target gene (10, 12). Recent studies of real-time PCR amplification have shown that low numbers of mismatches in the primer binding regions resulted in a quantification error up to 63.12% (13). Mainly, a conflicting result between real-time PCR and the reference method, direct immuno-fluorescence assay, was reported in which the mismatches in primer and probe binding region have affected real-time PCR accuracy (14). The real-time PCR assays developed in recent years have been commonly used due to their efficiency, high sensitivity and specificity. These assays have been adopted for routine diagnosis in national and reference laboratories (5, 10, 15). Most of them have been designed depend on the VP72-coding region, a highly conserved gene coding the major viral structure protein (8, 10). However, there are relatively few detection methods that target other genes. According to the analysis of genetic variation of the virus, we have recently found several mismatches between the OIE-recommended primers and multiple ASFV isolates (16). Due to new viral isolates displaying new genetic patterns, it is necessary to update the current real-time PCR assay to be able to detect all genotypes of the currently prevalent ASFV.

The *A137R* gene of ASFV encodes p11.5 protein, which is an important structural protein of ASFV. The high abundance expression of p11.5 protein in the three susceptible cell lines (WSL-HP, HEK 293, and Vero cells) infected with ASFV indicates that it plays an important role in the virus replication cycle (17). *A137R* gene is also highly expressed in Vero cells infected with ASFV according to transcriptome analysis reported by Cackett et al. (18). Considering the high expression level of the *A137R* gene, it is likely important throughout infection, which makes it an interesting candidate detection target. In this study, we developed and evaluated a SYBR Green-based real-time PCR assay for detection of ASFV which targeted the *A137R* gene. A comparative analysis between the *A137R* gene-based real-time PCR assay and OIE-recommended TaqMan PCR assay was performed. The results indicated that the *A137R* gene-based real-time PCR had higher sensitivity and advantages of saving time, labor and cost, which is a valuable feature for diagnosis and surveillance of ASFV infections among the pigs. This method will be beneficial for the prevention and control of ASF.

## Materials and Methods

### Virus and Nucleic Acid Extraction

The full length of *A137R* gene (414 bp) of ASFV genome (Pig/HLJ/2018, GenBank Accession Number MK333180) were synthetized by Sangon Biotech (Shanghai, China). Classical swine fever virus (CSFV), porcine reproductive and respiratory syndrome virus (PRRSV), porcine epidemic diarrhea virus (PEDV), porcine transmissible gastroenteritis virus (TGEV) and porcine rotavirus (PoRV) were obtained from our laboratory. Porcine circovirus 2 (PCV2) genome was a kind gift from professor Shaobin Shang at the College of Veterinary Medicine, Yangzhou University (Yangzhou, Jiangsu, China). The ASFV genome (GenBank accession number MH766894) was a kind gift from professor Rongliang Hu (Institute of Military Veterinary Medicine, Academy of Military Medical Sciences, Changchun, China). DNA was extracted by a FastPure Cell/Tissue DNA Isolation Mini Kit (Vazyme Biotech Co., Ltd., Nanjing, China). Total RNA was extracted with a FastPure Cell/Tissue Total RNA Isolation Kit (Vazyme Biotech Co., Ltd., Nanjing, China). Reverse transcription of RNA into cDNA was conducted using the PrimeScript RT reagent Kit (TaKaRa, Dalian, China) following the manufacturer's instruction. To avoid template degradation, eluted nucleic acid was appropriately diluted, aliquoted and stored in −70°C freezer.

### Design of the Primers

**Figure 1:**
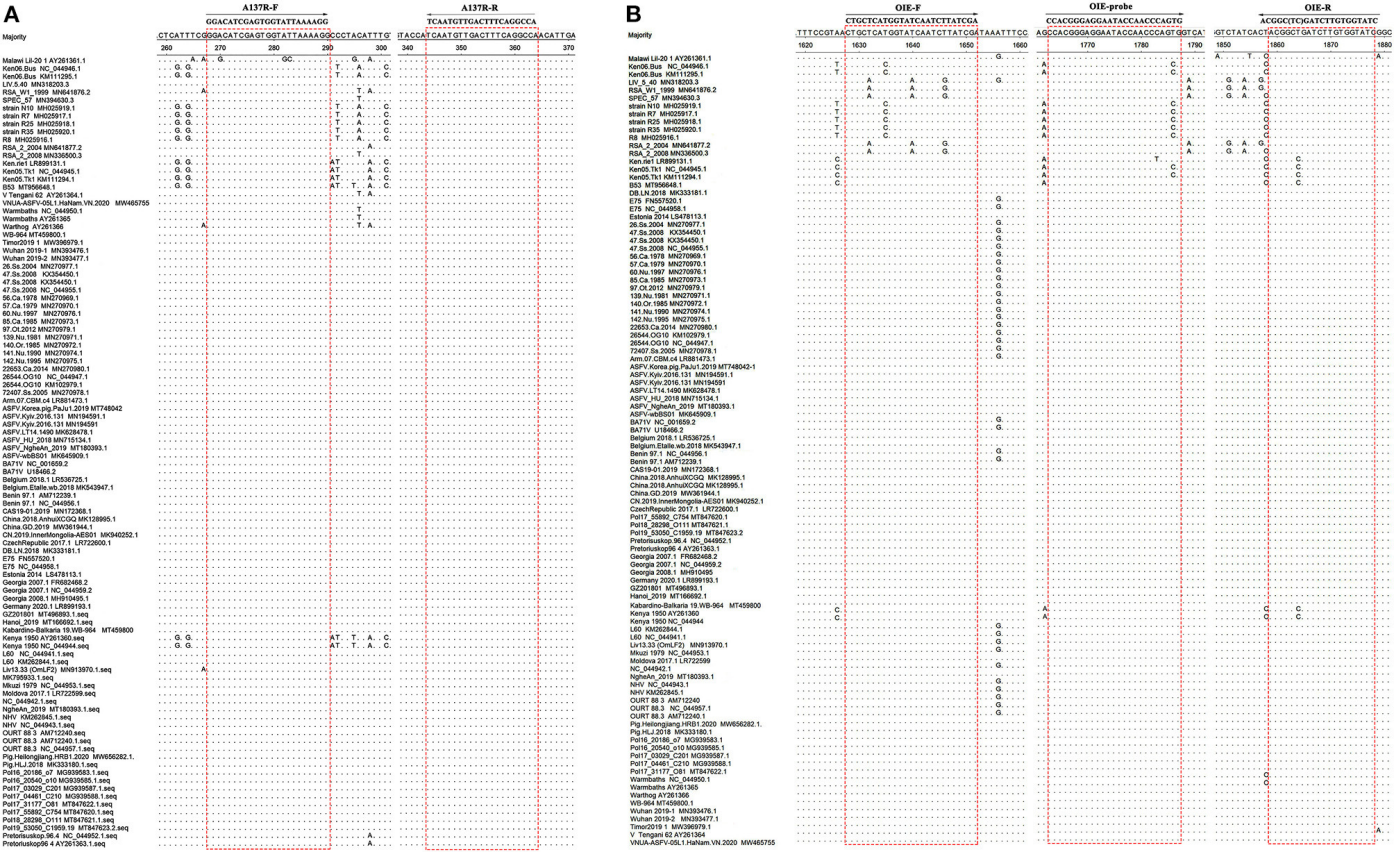
Alignment of sequences of the ASFV *A137R* gene and *B646L* gene in GenBank. **(A)** Locations of the target sequences of the primers *A137R*-F/*A137R*-R designed in the present study. **(B)** Locations of the target sites of the primers OIE-recommended TaqMan PCR method (19). Dots (.) indicate identical bases. The primer-binding sequences are boxed.

The forward primer *A137R*-F (5′-GGACATCGAGTGGTATTAAAAGG-3′, nt 250–272) and the reverse primer *A137R*-R (5′-TGGCCTGAAAGTCAACATTGA-3′, nt 326–346) were designed based on the alignment of 100 complete coding sequences (CDS) of the ASFV *A137R* gene available in the GenBank database (The Genbank accession numbers are shown in Figure 1). The sequences were analyzed using MegAlign and Protean software, version 7.1.0 (DNAstar). The primers were chosen in highly conserved regions of the targeted sequences, and the expected size of the real-time PCR product was 97 bp. The primers *A137R*-F/*A137R*-R corresponded to sites that were more conserved than those used in the OIE-recommended TaqMan PCR method (19) (Figure 1). To genotype ASFV in the samples that were positive in the developed assay, the forward primer *B646L*-F (5′-ATGGCATCAGGAGGAGCTTTTTG-3′, nt 1–23) and the reverse primer *B646L*-R (5′-TTAGGTACTGTAACGCAGCACAGC-3′, nt 1918–1941) were designed and used for automated dideoxynucleotide cycle sequencing of the *B646L* (p72) gene. All primers were synthesized by Sangon Biotech (Shanghai, China).

### Construction of Recombinant Standard Plasmid

The *A137R* gene was cloned into the TA vector pGEM-T easy (TaKaRa, Dalian, China) using common molecular biology techniques. The recombinant vector were transformed into *E. coli* DH5α competent cells (Vazyme Biotech Co., Ltd., Nanjing, China). Plasmid constructs were confirmed by Sanger sequencing (Sangon Biotech, Shanghai, China), and quantified by a NanoDrop ND-1000 Spectrophotometer (NanoDrop, Wilmington, Delaware, USA). Moreover, the standard plasmid copy number (N) was calculated by the following formula: *N* (*copies*/*ml*) = [6.02 × 10^23^ (*copies*/*mol*) × *C* (*g*/*ml*)]/*MW* (*g*/*mol*), where C is the concentration, and MW is the average molecular weight (double-stranded DNA) calculated as the number of bases (B) ×660 (g/mol) bases. B was determined by adding the number of bases in the plasmid and in the insert.

### Optimization of Real-Time PCR

The real-time PCR assay was developed and validated using the LightCycler (Roche Diagnostics, Mannheim, Germany) and TaKaRa TB Green Premix Ex Taq II (TaKaRa, Dalian, China). With other factors constant, the concentration of primers and annealing temperature were optimized to make the method more specific and sensitive (20). Four concentrations of the primer: 0.1, 0.2, 0.4, 0.6 μM and four different annealing temperatures: 56, 58, 60, 62°C were used in the experiment. According to the results, the optimum conditions were determined.

### Standard Curve

The standard plasmid was diluted 10-fold serially and amplified with the optimized real-time PCR system at the concentration of 1.0 × 10^8^−1.0 × 10^1^ copies/μL. The final standard curve is generated based on the CT value and the logarithm of standard copy number. Meanwhile, nuclease free water was used as negative control.

### Sensitivity

To determine the sensitivity of the assay, the standard plasmid was diluted 10-fold serially with concentrations ranging from 1.0 × 10^8^ to 1.0 × 10^1^ copies/μL. Prepared standards were amplified with the optimized real-time PCR system to investigate the sensitivity of the method. In addition, one of ASFV DNA samples with 10-fold dilution was tested using both *A137R* gene-based real-time PCR assay and the OIE-recommended TaqMan PCR assay developed by King et al. to compare the detection limit of the assays (19).

### Specificity

The real-time PCR assay was performed with other templates from PCV2, CSFV, PRRSV, PEDV, TGEV, and PoRV to evaluate the specificity of the reaction.

### Repeatability

The standard plasmids with 10-fold serial dilution from 1.0 × 10^8^ to 1.0 × 10^1^ copies/μL were used for the repeatability test. The diluted standard plasmids were stored in −70°C freezer immediately after each dilution was made. For the intra-assay test, three replicate samples from each dilution were tested in the same run. For the inter-assay test, each dilution of standard plasmids was tested in three independent runs to measure the test reliability or reproducibility regarding the mean CT-values with standard deviation (SD) and coefficient variation (CV).

### Clinical Sample Detection

A total of 34 specimens, including 10 swine anticoagulant blood, 10 tissue samples, 10 cloacal swabs and 4 samples of pig livers were processed for DNA extraction. Four samples of pig livers were collected from different supermarkets in Yangzhou, China. Other specimens were a kind gift from professor Rongliang Hu (Institute of Military Veterinary Medicine, Academy of Military Medical Sciences, Changchun, China). All the samples were stored at −70°C for subsequent use. DNA extracted from each of specimens was detected by *A137R* gene-based real-time PCR assay. The results were compared with those obtained by OIE-recommended TaqMan PCR that was performed in parallel. The samples with different results were sequenced and analyzed using primers *B646L*-F/*B646L*-R. Subsequently, 7 selected DNA of ASFV positive specimens, which included 2 swine anticoagulant blood, 3 tissue samples and 2 cloacal swabs were diluted with serial 10-fold dilutions. These dilution series DNA were tested both by the developed method and the OIE-recommended TaqMan PCR.

## Results

### Optimization of Real-Time PCR System

According to the results of Orthogonal test, the concentration of primers at 0.4 μM was the most efficient. The optimized real-time PCR reaction volume was 20 μL containing 10 μL TB Green Premix Ex Taq II, 0.4 μM forward primer, 0.4 μM reverse primer, 6.4 μL RNase Free ddH_2_O and 2.0 μL DNA template. The optimal program was as follows: one cycle of 30 s at 95°C, 45 cycles of 5 s at 95°C and 30 s at 62°C. Fluorescence signals for each sample were harvested at the end of each step at 62°C. Finally, the dissolution curve was added: one cycle at 95°C for 15 s, 60°C for 30 s and 95°C for 15 s.

### Establishment of Standard Curve

**Figure 2:**
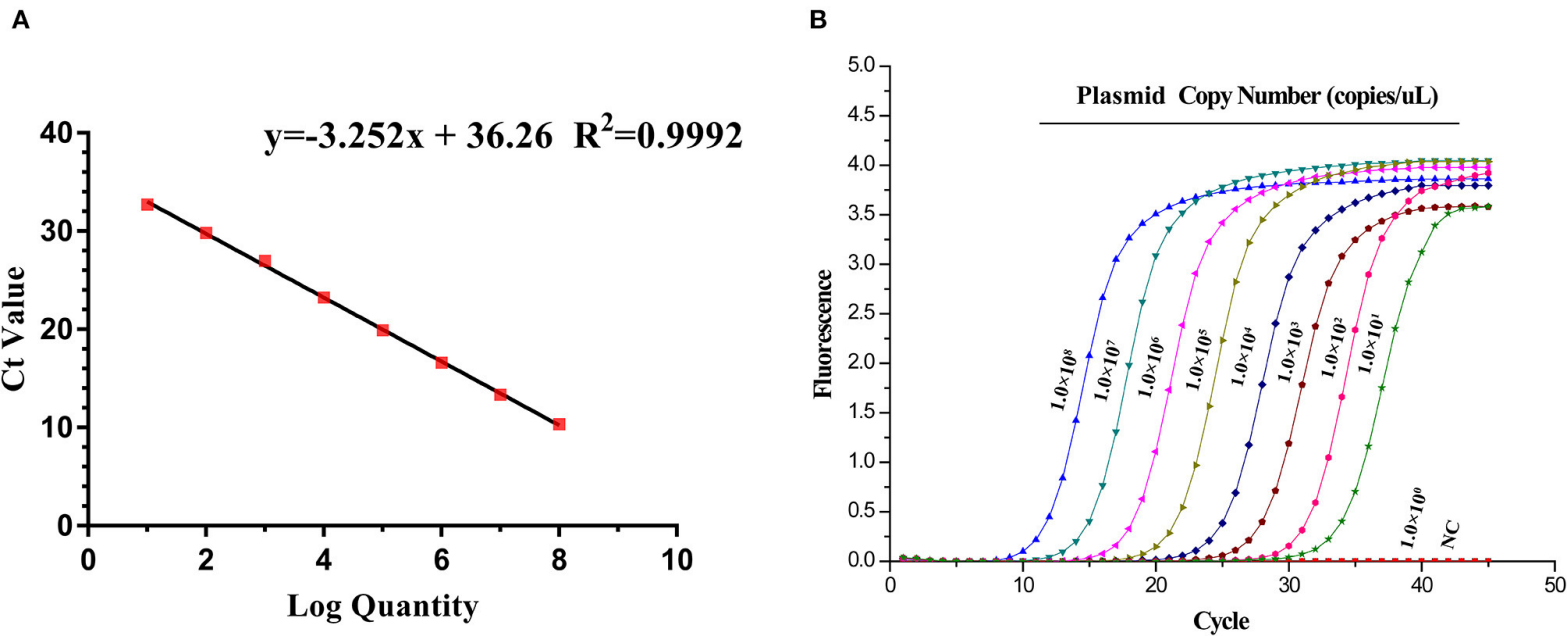
Standard curve and sensitivity tests for *A137R* gene-based real-time PCR assay of ASFV. **(A)** Establishment of standard curve for *A137R* gene-based real-time PCR. The 10-fold serial dilutions ranging from 1.0 × 10^8^ to 1.0 × 10^1^ copies/μL of DNA plasmid were tested in the real-time PCR. Each point corresponds to the mean value of three replicates. The optimal standard formula is y = −3.252x + 36.26, and the correlation coefficient is 0.9992. **(B)** Sensitivity tests for *A137R* gene-based real-time PCR assay of ASFV. Ten-fold serial dilutions of the DNA plasmid were used to perform the real-time PCR to obtain the expanded curve of the assay.

To construct a standard curve with the logarithm of plasmid copy numbers and the measured CT value (Figure 2A), serial plasmid dilutions at the concentration of 1.0 × 10^8^-1.0 × 10^1^ copies/μL were prepared. Three replicates were tested for each dilution. The optimal curve was selected as the standard curve. Establishment of ASFV standard curve with abscissa as logarithm of copy number and ordinate as CT-value, the correlation coefficient (*R*^2^) was 0.9992, the slope was −3.252, and the intercept was 36.26. The standard formula is y = −3.252x + 36.26, and *R*^2^ = 0.9992. Moreover, negative or extraction blank controls should have a CT > 38.0.

### Sensitivity of Real-Time PCR Targeting the *A137R* Gene

**Figure 3:**
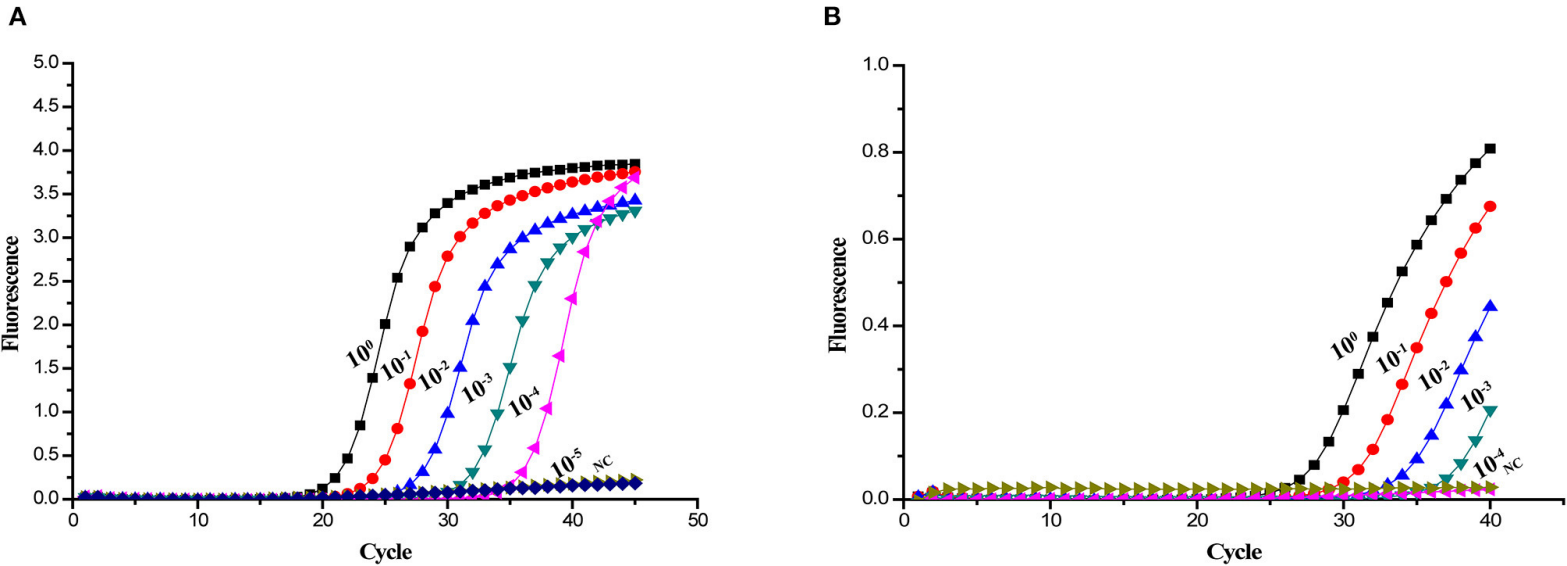
Sensitivity of different real-time PCR assays for detection of ASFV DNA. Ten-fold serial dilutions of the ASFV DNA were used to perform the real-time PCR to obtain the expanded curve of the assays. **(A)** Detection limit of the *A137R* gene-based real-time PCR assay. **(B)** Detection limit of the OIE-recommended TaqMan PCR assay.

Sensitivity tests were performed using plasmids ranging from 1.0 × 10^8^ to 1.0 × 10^1^ copies/μL. The 10-fold serial dilutions of the standard plasmids were simultaneously detected by the established real-time PCR. The minimum detection template concentration of method was 10 copies/μL (Figure 2B). As shown in Figure 3, the *A137R* gene-based real-time PCR had 10 times lower detection limit when compared with that of the OIE-recommended TaqMan PCR (19), indicating that the established real-time PCR was more sensitive than OIE-recommended TaqMan PCR.

### Specificity of Real-Time PCR Targeting the *A137R* Gene

**Figure 4:**
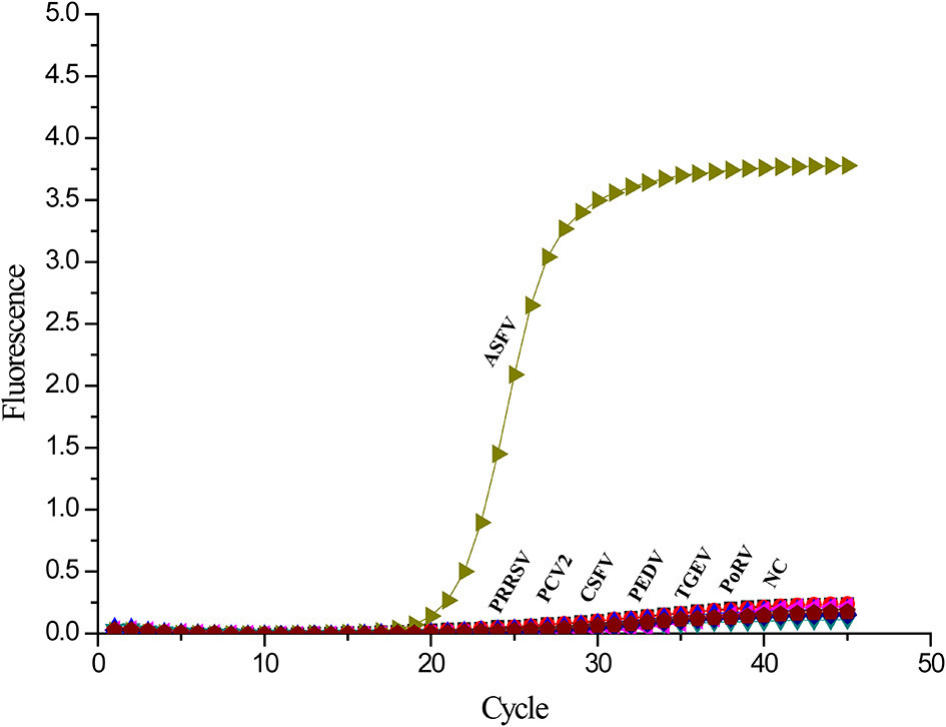
Specificity analysis of the *A137R* gene-based real-time PCR. Only ASFV showed a positive fluorescence signal, and no positive signal was observed with other swine viruses.

To investigate the specificity of the real-time PCR, other templates from common swine viruses were tested with the assay. The ASFV DNA samples got strong fluorescent signals. However, the negative control and other swine viruses such as PCV2, CSFV, PRRSV, PEDV, TGEV, and PoRV showed no signal amplification (Figure 4). Therefore, there is a significant distinction between ASFV and other swine viruses by comparing the signal strength at different levels.

### Repeatability of Real-Time PCR Targeting the *A137R* Gene

The intra-assay repeatability was assessed by testing 10-fold serial dilutions of the standard plasmids ranging from 1.0 × 10^8^ to 1.0 × 10^1^ copies/μL in three replicates. To determine the intre-assay repeatability, the same standard plasmids were analyzed in triplicate on 3 different days. In result, intra-assay standard deviations (SD) ranged from 0.02 to 0.34, and inter-assay standard deviations (SD) ranged from 0.04 to 0.32. The coefficient of variation (CV) value of intra-assay and inter-assay were 0.12–0.99% and 0.26–0.96%, respectively (Table 1). The results showed that the real-time PCR targeting the *A137R* gene developed in this study is repeatable.

**Table 1:** The repeatability of *A137R* gene-based real-time PCR.

**Reference**	**Intra-assay repeatability of CT-value**			**Inter-assay repeatability of CT-value**		
**copies/μL**	**Mean**	**SD**	**CV (%)**	**Mean**	**SD**	**CV (%)**
1 ×10^8^	11.00	0.02	0.23	11.03	0.05	0.46
1 ×10^7^	14.12	0.05	0.32	14.12	0.04	0.26
1 ×10^6^	17.38	0.02	0.12	17.47	0.12	0.67
1 ×10^5^	20.61	0.03	0.14	20.70	0.11	0.52
1 ×10^4^	23.95	0.09	0.37	24.20	0.18	0.75
1 ×10^3^	26.82	0.12	0.45	26.38	0.10	0.36
1 ×10^2^	30.24	0.27	0.90	30.22	0.29	0.96
1 ×10^1^	33.72	0.34	0.99	34.35	0.32	0.94
Mean		0.12	0.44		0.15	0.62

### Clinical Sample Application

To determine the practical application of this method, we used 34 specimens to compare the real-time PCR targeting the *A137R* gene and recommended TaqMan PCR. The positivity rates of ASFV in swine anticoagulant blood, tissue samples and cloacal swabs were determined to be 90% (9/10), 100% (10/10), and 80% (8/10) by OIE-recommended TaqMan PCR. While, they were 100% (10/10) of swine anticoagulant blood, 100% of tissue samples (10/10) and 90% (9/10) of cloacal swabs in the *A137R* gene-based real-time PCR assay. Four samples of pig livers from supermarket were detected negative by both methods. As shown in Table 2, the positivity rate of the *A137R* gene-based real-time PCR was 85.29% (29/34) and only 79.41% (27/34) in the OIE-recommended TaqMan PCR. The positive samples in this method but negative in the OIE-recommended method were sequenced. Sequencing results showed that these sequences are identical to the genotype II viruses circulating in China, without any mutations. The positive detection rate of these clinical samples tested by our assay was higher than that of OIE-recommended TaqMan PCR, which may correlate with different expression level of target genes in early infection. The results of 7 selected DNA of ASFV positive specimens in Table 3 showed that *A137R* gene-based real-time PCR had an equal or 10 times higher sensitivity when compared with the OIE-recommended TaqMan PCR. Therefore, the method developed in this study showed better performance than OIE-recommended TaqMan PCR assay in detecting ASFV gene from clinical samples.

**Table 2:** Comparison of detecting clinical samples by *A137R* gene-based real-time PCR and OIE-recommended TaqMan PCR.

		***A137R*** **gene-based**		**Summary**
		**real-time PCR**		
		**Positive**	**Negative**	
OIE-recommended	Positive	27	0	27
TaqMan PCR	Negative	2	5	7
Summary		29	5	34

**Table 3:** Sensitivity of different PCR assays for detection of ASFV.

**Specimens**	**Detection limit**	
	***A137R* gene-based**	**OIE-recommended**
	**real-time PCR (CT value)**	**TaqMan PCR (CT value)**
Anticoagulant blood 1	10^−3^ (32.57)	10^−2^ (33.30)
Anticoagulant blood 2	10^−1^ (34.29)	10^−1^ (34.90)
Tissue sample 1	10^−4^ (35.49)	10^−3^ (36.97)
Tissue sample 2	10^−4^ (33.75)	10^−3^ (36.69)
Tissue sample 3	10^−5^ (32.87)	10^−4^ (32.60)
Cloaca swab 1	10^−1^ (34.42)	10^−1^ (34.78)
Cloaca swab 2	10^−2^ (33.75)	10^−1^ (32.33)

*The detection limit is given as the lowest detectable dilution of the DNA*.

## Discussion

African swine fever is a highly contagious disease with 100% morbidity and mortality. China, the largest pork producer and consumer, had attracted global concerns because of the prevalence of ASFV (21). Various ASFV vaccine strategies focusing on DNA-, antigen- and virus vector-based have been investigated. However, there is still no successful vaccine until now (22). Many control measures were conducted by Chinese government to inhibit the rapid spread of ASFV which emphasize the need for early, rapid, high rates of sensitive and specific diagnosis in ASFV. For this purpose, PCR assays have been recommended as the methods for detecting ASFV.

Real-time PCR and conventional PCR assay are the most widely used assay and also recommended by OIE for virological and molecular diagnosis of ASF. However, one report showed that the OIE-recommended conventional PCR for ASFV had low sensitivity, most likely due to an imperfect match of the primers with the target sequences of some ASFV genotypes (5, 10). Although OIE-recommended TaqMan PCR has shown excellent sensitivity and specificity rates, the high fidelity of the method is slightly decreased when the samples showed low or weak ASFV-positive (23, 24). Therefore, it is necessary to find more effective detection targets to improve the detection of ASFV. Some scholars have analyzed ASFV protein expression in three susceptible mammalian cell lines representing a susceptible host (wild boar) and two non-susceptible species (human and green monkey) by mass spectrometry. The results showed that p11.5 protein encoded by *A137R* gene was highly expressed in three susceptible cell lines infected with ASFV (17). The results are consistent with transcriptome analysis (18). It is speculated that p11.5 protein may play an important role in the viral replication cycle. Due to SYBR Green-based real-time PCR have similar sensitivity and specificity as TaqMan-based PCR (25), we developed and evaluated a SYBR Green-based real-time PCR assay which target the *A137R* gene for detection of ASFV.

In present study, a pair of primers was designed based on the alignment of *A137R* gene sequences targeting conserved areas of the gene and making these primers more universally applicable than those used in OIE-recommended TaqMan PCR. BLAST searches of the new ASFV primers confirmed that the primer sequences target highly conserved regions of the ASFV *A137R* gene sequences. Only one ASFV isolate was found to have mismatches with the primers used in the new real-time PCR, while several mismatches were found between the OIE-recommended primers and multiple ASFV isolates. These indicated that the novel assay could be used for universal detection of ASFV. After optimization, the *A137R* gene-based real-time PCR assay was established. A correlation coefficient higher than 0.98 indicates that the linear correlation of the standard curve was good (26). The results showed that the correlation standard curve equation was y = −3.252x + 36.26, and the correlation coefficient was 0.9992. Therefore, the standard curve showed a good linear correlation. Based on the data, the detection limit of *A137R* gene-based real-time PCR was as low as 10 copies/μL. No positive signal was observed with other swine viruses, indicating favorable specificity of the assay. Repeatability analysis revealed the intra-assay and inter-assay CVs to be lower than 1%, which indicated the repeatable of the method. DNA of ASFV specimens was tested after 10-fold serial dilution. The results showed that *A137R* gene-based real-time PCR was more sensitive than OIE-recommended TaqMan PCR in most ASFV specimens. When testing swine anticoagulant blood and cloacal swabs with lower virus levels, the positivity rates of the *A137R* gene-based real-time PCR was higher than OIE-recommended TaqMan PCR. Recently, genotype I ASFV has been reported in domestic pigs in China (27). The *A137R* primers used in the developed SYBR Green-based real-time PCR targeting the *A137R* gene of ASFV are highly conserved across all known ASFV genotypes, including genotype I, making it possible to detect both genotype I and II ASFV co-circulating in China at the moment. The real-time PCR assay developed in this study enables the detection of virus in the early stage of infection or low virus concentration, providing a good method for the early diagnosis of the disease.

In conclusion, our data indicated that SYBR Green-based real-time PCR targeting the *A137R* gene is a sensitive, specific, repeatable and reliable method to detect ASFV. In addition, SYBR Green-based method has advantages of saving time, labor, and cost. It could therefore be a promising tool for clinical diagnosis and epidemiological investigation of ASFV as well as for future research on the virus.

## Data Availability Statement

The raw data supporting the conclusions of this article will be made available by the authors, without undue reservation.

## Author Contributions

Material preparation, data collection, and analysis were performed by DY, RG, HL, CB, KQ, and AQ. The first draft of the manuscript was written by DY and revised by AQ and KQ. All authors contributed to the study conception and design, commented on previous versions of the manuscript, and read and approved the final manuscript.

## Funding

This work was supported by National Science Foundation of China (31941016), the National Key Research and Development Program (Grant No. 2021YFD180001), the Priority Academic Program Development of Jiangsu Higher Education Institutions, and the Jiangsu Co-innovation Center for the Prevention and Control of Important Animal Infectious Diseases and Zoonoses. The funding bodies did not play direct roles in the design of the study and collection, analysis, and interpretation of data and in writing the manuscript.

## Conflict of Interest

The authors declare that the research was conducted in the absence of any commercial or financial relationships that could be construed as a potential conflict of interest.

## Publisher's Note

All claims expressed in this article are solely those of the authors and do not necessarily represent those of their affiliated organizations, or those of the publisher, the editors and the reviewers. Any product that may be evaluated in this article, or claim that may be made by its manufacturer, is not guaranteed or endorsed by the publisher.
